# Whole exome sequencing and DNA methylation analysis in a clinical amyotrophic lateral sclerosis cohort

**DOI:** 10.1002/mgg3.302

**Published:** 2017-06-12

**Authors:** Fleur C. Garton, Beben Benyamin, Qiongyi Zhao, Zhijun Liu, Jacob Gratten, Anjali K. Henders, Zong‐Hong Zhang, Janette Edson, Sarah Furlong, Sarah Morgan, Susan Heggie, Kathryn Thorpe, Casey Pfluger, Karen A. Mather, Perminder S. Sachdev, Allan F. McRae, Matthew R. Robinson, Sonia Shah, Peter M. Visscher, Marie Mangelsdorf, Robert D. Henderson, Naomi R. Wray, Pamela A. McCombe

**Affiliations:** ^1^ Queensland Brain Institute The University of Queensland St Lucia Queensland 4072 Australia; ^2^ Institute for Molecular Bioscience The University of Queensland St Lucia Queensland 4072 Australia; ^3^ Reta Lila Weston Institute UCL Institute of Neurology London UK; ^4^ Department of Molecular Neuroscience UCL Institute of Neurology London UK; ^5^ UQ Centre for Clinical Research The University of Queensland Royal Brisbane & Women's Hospital Brisbane 4029 Australia; ^6^ Centre for Healthy Brain Ageing School of Psychiatry Faculty of Medicine The University of New South Wales Sydney New South Wales 2052 Australia; ^7^ Neuropsychiatric Institute Prince of Wales Hospital Randwick New South Wales Australia; ^8^ University of Queensland Diamantina Institute Translational Research Institute Brisbane Queensland 4012 Australia; ^9^ Department of Neurology Royal Brisbane & Women's Hospital Brisbane 4029 Australia

**Keywords:** ALS, clinical genetics, motor neuron disease, next‐generation sequencing, whole exome sequencing

## Abstract

**Background:**

Gene discovery has provided remarkable biological insights into amyotrophic lateral sclerosis (ALS). One challenge for clinical application of genetic testing is critical evaluation of the significance of reported variants.

**Methods:**

We use whole exome sequencing (WES) to develop a clinically relevant approach to identify a subset of ALS patients harboring likely pathogenic mutations. In parallel, we assess if DNA methylation can be used to screen for pathogenicity of novel variants since a methylation signature has been shown to associate with the pathogenic *C9orf72* expansion, but has not been explored for other ALS mutations. Australian patients identified with ALS‐relevant variants were cross‐checked with population databases and case reports to critically assess whether they were “likely causal,” “uncertain significance,” or “unlikely causal.”

**Results:**

Published ALS variants were identified in >10% of patients; however, in only 3% of patients (4/120) could these be confidently considered pathogenic (in *SOD1* and *TARDBP*). We found no evidence for a differential DNA methylation signature in these mutation carriers.

**Conclusions:**

The use of WES in a typical ALS clinic demonstrates a critical approach to variant assessment with the capability to combine cohorts to enhance the largely unknown genetic basis of ALS.

## Introduction

Amyotrophic lateral sclerosis (ALS) is a fatal neurodegenerative disease characterized by the progressive loss of upper and lower motor neurons with diagnosis based on clinical and neurophysiological criteria. Typically, it presents as late‐onset muscle weakness leading to paralysis and death within 3–5 years (Hardiman et al. 2011; Majounie et al. 2012), although there is considerable heterogeneity of motor and extramotor features (McCombe et al. 2016).

Amyotrophic lateral sclerosis likely arises from a combination of genetic susceptibility (Marangi and Traynor 2015; Renton et al. 2014) and environmental factors (Fang et al. 2009), but the contributing genetic variants can differ among individuals. About 10% of patients report a first degree relative with a diagnosis of ALS (familial ALS, fALS). Analyses of genetic data from large samples of fALS cases and nonaffected family members have led to the identification of mutations that segregate with disease, and these are collated in the ALS online Database (ALSoD) (Abel et al. 2012). Some recorded variants are pathogenic, or at least highly penetrant, and the latter include the *C9orf72* hexanucleotide (G_4_C_2_) repeat expansion (HRE) (DeJesus‐Hernandez et al. 2011a; Renton et al. 2011a), the most common variant known to cause ALS (MIM#105550). Worldwide, the *C9orf72* HRE is estimated to account for ~38% fALS and ~6% of those presenting as sporadic (sALS, i.e., no or limited family history) with penetrance estimated as 50% by 58 years, and almost 100% by 80 years (Majounie et al. 2012). Three other genes (*SOD1*,* TARDBP*, and *FUS*) harbor pathogenic variants which together account for ~20% of fALS cases (Renton et al. 2014) (MIM#105400, #612069, #608030, respectively). In total, reported variants that fulfill adequate objective criteria for causation (Abel et al. 2013; MacArthur et al. 2014; Stenson et al. 2014) are in ~30 genes (Morgan et al. 2015). Most of these are autosomal dominant variants located in exonic regions of protein coding genes, however, many ALS‐associated variants, even those in well‐known genes, have not been sufficiently confirmed as pathogenic (Eisen et al. 2008). In fact, approximately 100 additional genes (658 known variants) are listed in ALSoD (Abel et al. 2012); the list includes rare variants requiring further evidence for proof of causality, as well as common variants that increase the risk of ALS. As discovery sample sizes increase, more variants are expected to be classified as pathogenic, while other variants in the database may be confirmed as false positives (benign). False positive reporting for variants segregating with disease is well recognized; for example, on average, each of the >60K individuals in the Exome Aggregation Consortium (ExAC) (Lek et al. 2016) study harbors 54 variants listed as causative of disease in the Mendelian disease database (OMIM). Moreover, larger sample sizes have allowed more accurate estimates of population frequencies of OMIM variants showing that 80% of them are too common to be compatible with disease pathogenicity.

With decreasing costs of genotyping technologies, implementation of routine genetic diagnostic testing in the ALS clinic is becoming more achievable and is desirable because earlier diagnosis could be achieved for a subset of individuals and a genetically informed diagnosis may allow better stratification of patients for therapy. For those presenting with sALS, ~11% harbor pathogenic variants (Renton et al. 2014). While changes in variant identification rely on the underlying etiologies of ALS, this proportion may increase as discovery samples increase to allow the list of confirmed variants to grow. Genetic testing for known causative mutations can be performed as single variant tests, or as a multiple variant test in a targeted capture, or through whole exome or genome sequencing (WES/WGS). The WES/WGS approach allows interrogation of the data for known mutations, as well as novel variants in causative genes, with periodic reanalysis of the data as new results become available. Effective filtering strategies with large control datasets are required to maximize true positives while reducing false positives. Other genetic technologies, such as DNA methylation studies, could contribute to predicting the pathogenicity of novel variants (Bonder et al. 2017). For example, carriers of a pathogenic HRE expansion in the *C9orf72* gene have been reported to carry a *C9orf72* methylation signature (Xi et al. 2013; He et al. 2015; Gijselinck et al. 2016). This phenomenon has not been explored for other ALS mutations.

Here, we set out to determine what conclusions could be drawn from a typical ALS clinic if all patients were put forward for generation of WES. We propose a filtering strategy to highlight relevant results to cover all modes of variant architecture and assess whether a methylation signature coexists with causal mutations.

## Materials and Methods

### Ethical compliance

The study was approved by the RBWH, University of Queensland, QIMR, and UNSW Research Ethics Committees.

### Subjects

Australian ALS patients were recruited from the Royal Brisbane and Women's Hospital (RBWH) and were defined as definite or probable ALS according to the revised El Escorial criteria (Brooks et al. 2000) (*N* = 120). For the methylation capture, patients were recruited as above with healthy controls sourced from Brisbane and Sydney across three different sites: RBWH, Queensland Institute of Medical Research (QIMR), and University of New South Wales (UNSW) (see Supporting Information).

### Genetic data

#### Whole exome sequencing and genome‐wide methylation

Briefly, patient DNA was extracted from fresh whole blood using manual extraction protocols. In controls obtained from UNSW, the majority (90%) of DNA was extracted from frozen whole blood or lymphocytes using a kit (Qiagen Autopure, Hilden, Germany). Sequencing libraries were prepared using the KAPA HYPER Prep kit (KAPA Biosystems, Wilmington, MA, USA) and followed by targeted exome capture using the Nimblegen SeqCap EZ Exome v3 (64 Mb) kit (Roche, Madison, WI, USA), both according to the manufacturer's instructions. Patient samples were sequenced in a 126‐bp paired end mode using the Illumina HiSeq 2000 platform. Processing and sequence alignment were by standard protocols (GATK pipeline) (McKenna et al. 2010), and variants were annotated using the ANNOVAR software tool (version 2015 Jun17) (Wang et al. 2010). Following variant call and annotation, standard quality control (QC) steps were implemented, that is, common SNPs to remove individuals identified as being discordant for sex, excessive heterozygosity, or relatedness. Genome‐wide methylation data were generated using the Illumina HumanMethylation450 array, which contains probes covering 99% of reference sequences (RefSeq) genes and 96% of CpG islands. A standard QC pipeline was applied to remove probes bound to multiple locations and duplicate individuals (Supporting Information).

#### WES variant filtering and statistical analysis

**Figure 1:**
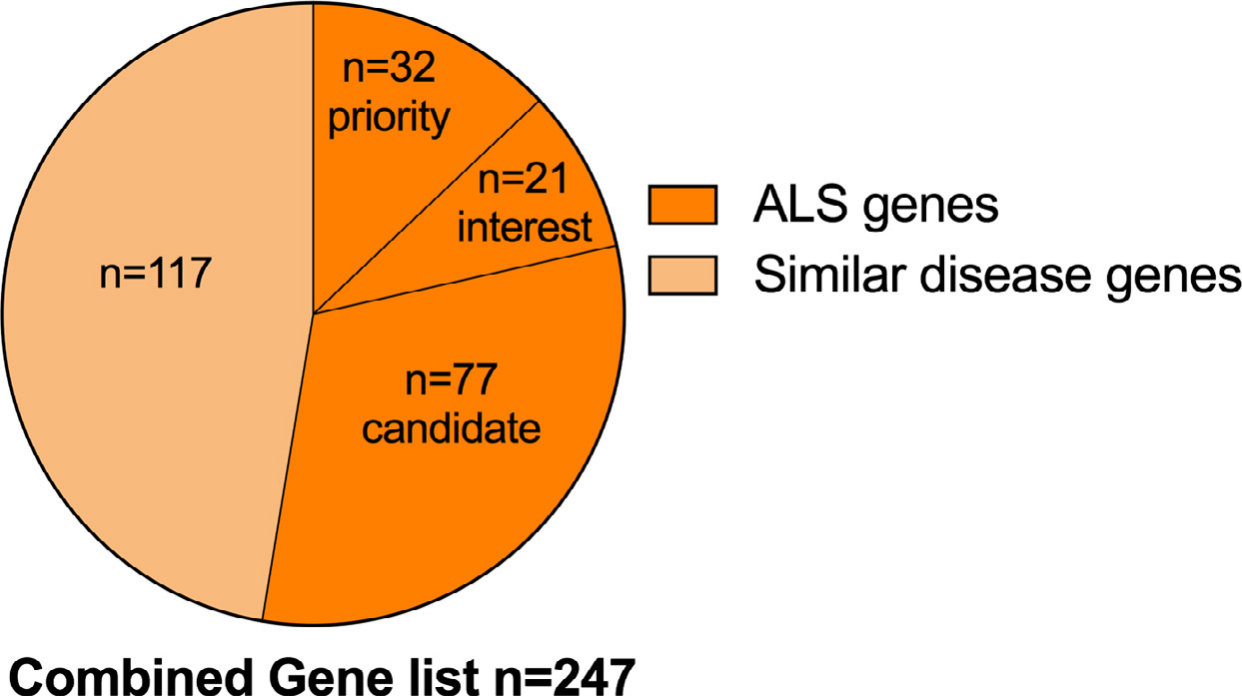
Combined gene list (*n* = 247) used for variant filtering. Genes associated with ALS were manually curated from ALSoD and HGMD and divided into independent subgroups (priority, interest, candidate). Also considered were genes with a high level of evidence for pathogenicity in diseases showing evidence of clinical overlap with ALS (frontotemporal dementia, spinal muscular atrophy, spinal bulbar muscular atrophy, distal hereditary motor neuropathy, Charcot–Marie–Tooth disease, hereditary spastic paraplegia, hereditary ataxia, and distal myopathy). Gene lists are found in Table S1.

The pathogenicity of single‐nucleotide variants was evaluated based on a number of filtering steps (Supporting Information). Briefly, known variants and relevant genes were compiled from two databases, the human gene mutation database (HGMD) (Stenson et al. 2014) and the amyotrophic lateral sclerosis online database (ALSoD) (Abel et al. 2012). This consisted of three reference lists to examine patient exomes for known variants or novel variants in relevant genes (Fig. 1; Table S1), these lists were:

1. Variants associated with ALS (*n* = 1158 variants)
2. Variants in known ALS genes characterized based on literature review as priority (*n* = 32 genes), interest (*n* = 21 genes), and candidate (*n* = 77 genes). Pathogenic insertion and deletions (indels) were analyzed in a subset of genes (*n* = 21 genes, Table S1) previously published to contain indels or loss‐of‐function mutations known to cause ALS symptoms.
3. Variants in related disease genes, that is, genes with evidence of causation for diseases with genetic/symptomatic links to ALS, including frontotemporal dementia, spinal muscular atrophy, spinal bulbar muscular atrophy, distal hereditary motor neuropathy, Charcot–Marie–Tooth disease, hereditary spastic paraplegia, hereditary ataxia, and distal myopathy (HGMD, *n* = 117 genes).

These lists were hierarchical, that is, variants filtered in list (1) were not included in list (2).

Based on the latest recommendations of ExAC, novel variants were filtered out if their minor allele frequency (MAF) in any of the 15 available reference populations (Supporting Information) was above the specified threshold (Lek et al. 2016). This is referred to as the “population maximum” or “popmax” approach, whereby a variant is considered unlikely to be pathogenic if it has achieved the threshold frequency of ≥0.00005 for autosomal dominant and ≥0.01 for recessive/compound heterozygous candidate variants, respectively (Cirulli et al. 2015), in any ancestry‐specific population. This applies a more stringent threshold than using an average frequency across all populations to reduce the number of false positives. The final list of variants that passed the popmax filter included splice site mutations and nonsynonymous variants predicted to be deleterious by a combined evaluation of 18 current deleteriousness scoring methods as implemented in MetaLR and MetaSVM (Dong et al. 2015). Variants identified in our patients were then cross checked with the literature and online databases for case reports to assess whether they were “likely causal,” “uncertain significance,” or in the case of a known variant with high minor allele frequency “unlikely causal.”

We were underpowered to perform either burden testing or gene‐set analyses; however, exome data will be uploaded to ALSdb (V2, New York City, New York [URL: http://alsdb.org]) to contribute to future larger studies. Likely causal pathogenic variants and anonymous patient data will be reported in the ALSoD (Abel et al. 2012) patient database.

### Detection of DNA methylation outliers

From previous analyses and published results, 40–60% of *C9orf72* HRE carriers have been shown to be extreme DNA methylation outliers in blood (Xi et al. 2013; He et al. 2015; Gijselinck et al. 2016). Since genome‐wide association studies have identified single‐ nucleotide polymorphisms (SNPs) associated with variation in DNA methylation between individuals (Bonder et al. 2017), our hypothesis was that carriers of pathogenic variants in known ALS genes may exhibit hypo‐ or hypermethylation in the same gene, relative to noncarriers. For an initial screening, carriers were considered outliers if they lay 1.5× outside the interquartile range of noncarriers. Analysis particularly focused on probes closest to the transcription start site (TSS) of the single‐nucleotide variant gene. More in‐depth analysis was not justified based on the results of this initial screening.

## Results

A summary of clinical data for the 120 unrelated patients is given in Table 1. Briefly, the cohort included 75 males and 45 females, median age of onset was 61 years (SD = 10.1) and survival was 2.9 years (SD = 2.4) (*n* = 80). For WES, ~741.48 Gbp of sequence data were generated for these individuals, with a mean coverage of 53.9× per individual. For DNA methylation analysis, 118 cases and 111 unrelated controls and 432,216 probes were used in analysis.

**Table 1:** Characteristics of ALS participants

Number of ALS cases	120
Number of males	75 (62.5%)
Population in clinic capture cohort^a^	3.68 million
European ancestry	113 (93.0%)
Number reported to have first degree relatives with ALS/FTD (i.e., parent/sibling)	10 (8.3%)
Number reported to have second/third degree relative with ALS/FTD (i.e., grandparent/avuncular/cousin) and no first degree relatives.	2 (1.6%)
Number reported a family history of related disease (dementia/MS/parkinsonism)	5 (4.1%)
Family history unknown	14 (11.5%)
Number of *C9orf72* HRE^b^ positive	9 (7.5%)
Number of slow progressing >7 years	18 (15.0%)
Median (range) age at onset (years)	61 (SD ±10.1)
Median years survival (at death or censoring, *n* = 80)	2.9 (SD ±2.4)
Onset location^c^	
Number bulbar onset	30/118 (25%)
Number upper limb onset	33/118 (28%)
Number lower limb onset	53/118 (45%)
Number bulbar + Lower limb onset	1/118 (1%)
Number bulbar + Upper limb onset	1/118 (1%)

SD, standard deviation.

a Population estimate based on Northern Rivers NSW shires and Southeast QLD shires.

b See Supporting Information for details.

c Two individuals did not have a specified onset location. Family history reflects subject self‐report of a family member known to have had ALS, FTD, or a related disease. Those that did not know their family history were reported as unknown.

### Filtering of WES variants against reference list 1: variants reported to be associated with ALS

From 120 ALS patients, 17 variants were listed in either ALSoD or HGMD databases.

Four subjects (4/120 = 3%) were found to harbor variants classified as likely pathogenic, including two variants in *SOD1*, p.Gly94Val and p.Ile114Thr, and two variants in the *TARDBP*, p.Met337Val and p.Ala382Thr (Tables 2 and S2). Clinical data for these patients and comparison to available clinical notes from previous reports are listed in the Supporting Information. Although the remaining 13 variants were listed in the databases, each of these had a popmax MAF of ≥0.002, which is incompatible with current estimates of ALS lifetime risk and therefore are unlikely to be causal (Table S3). Half of these variants were reported by Daoud et al. (2011) based on a candidate gene analysis that is likely to be underpowered for identification of causal rare variants (380 cases/controls). A *NEK1* variant identified in our cohort (p.Arg261His) increases the risk of ALS (OR=2.4, 95% CI 1.6–3.7) (Kenna et al. 2016) but is not fully penetrant. No autosomal recessive or compound heterozygous genotypes of the listed ALS variants were identified.

**Table 2:** Previously reported pathogenic variants found in Brisbane ALS patients

Gene	Exon, nucleotide, amino acid change	Positive number/negative number	Popmax	SNP	References
*SOD1*	exon4:c.281G>T:p.Gly94Val NM_000454.4 a	1/119	NA		Brown et al. (2012)
*SOD1*	exon4:c.341T>C:p.Ile114Thr NM_000454.4 a	1/119	NA	rs121912441	Brown et al. (2012), Rosen et al. (1993)
*TARDBP*	exon6:c.1009A>G:p.Met337Val NM_007375.3	1/119	NA	rs80356730	Tamaoka et al. (2010)
*TARDBP*	exon6:c.1144G>A:p.Ala382Thr NM_007375.3 a	1/119	NA	rs367543041	Borghero et al. (2014), Quadri et al. (2011)

Positive number/negative number = number of cases identified with the alternate allele compared to those cases tested with the reference allele. All changes are single allele changes (heterozygous). Popmax = population maximum; frequency of the alternate allele in available control populations. A SNP rs number means that the SNP has been previously reported, but NA for popmax frequency means that the SNP is not listed in the databases and hence is assumed to be very rare. NCBI reference sequence provided.

a Variant independently confirmed by an Australian National Association of Testing Authorities (NATA) accredited laboratory.

### Filtering of WES variants against reference list 2: variants in ALS‐associated genes

For this filtering step we excluded patients who were positive for the likely pathogenic *SOD1* (*n* = 2), *TARDBP* (*n* = 2), or *C9orf72* (*n* = 9) so that 107 patients were examined for novel variants identified in known ALS genes. From the initial variant output of 2490, ~89% variants were too common to cause ALS with 2% being rare exonic changes. Variants that were predicted to be damaging included six in genes we labeled “priority”, three in “interest” genes, and seven in “candidate” genes (Tables 3 and S4).

**Table 3:** Novel damaging variants in ALS genes found in Brisbane ALS patients

Gene	Exon, nucleotide, amino acid changes	Positive number/negative number	Popmax	SNP
*DCTN1*	exon19:c.2244C>G:p.Asp748Glu NM_004082.4	1/118	0.00003	–
*DCTN1*	exon14:c.1486G>C:p.Val496Leu NM_004082.4	1/84	0.00003	–
*FUS*	exon3:c.101G>A:p.Gly34Glu NM_004960.3	1/119	0	–
*NEFH*	exon4:c.2176G>C:p.Val726Leu NM_021076.3	1/119	0	–
*SPG11*	exon26:c.4557T>A:p.Asp1519Glu NM_025137.3	1/119	0.00001	–
*TBK1*	exon8:c.830delT:p.Leu277fs NM_013254.1	1/119	0	–

Positive number/negative number = number of cases identified with the alternate allele compared to those cases tested with the reference allele. All changes are single allele changes (heterozygous). Popmax = population maximum; frequency of the alternate allele in available control populations. NCBI reference sequence provided.

None of the variants had a reference SNP cluster ID (SNP).

Indel variants examined in a subset of 21 genes known to cause ALS or ALS symptoms including a frameshift deletion (not reported before) caused by a single base deletion in exon 8 (p.277) of the *TBK1* gene (Table 3). Using WES data to identify indels can produce high numbers of false positives (Fang et al. 2014) and Sanger sequencing is required for confirmation. If the deletion variant is confirmed, current evidence on the *TBK1* deletion variant profile across multiethnic ALS and/or FTD cohorts suggests this could be pathogenic (Cirulli et al. 2015; Oakes et al. 2017).

For recessive variants, the MAF filter (0.01) removed 60% of the variants. A further 25% were excluded as they were not exonic/missense or splice site variants. No subsequent recessive variants were identified in any patients.

### Filtering of WES variants against reference list 3: variants in related‐disease genes

A total of 2290 variants were identified in the 117 genes harboring causal variants for diseases identified to have genetic/symptomatic cross‐over with ALS (Renton et al. 2014). Ninety‐one percent of variants were removed using a MAF of 0.00005, with a further 7% and 1% subsequently removed for being outside exons or synonymous SNPs, respectively. Twenty‐five autosomal dominant changes were identified in ALS cases with 18 of these predicted to be damaging. One was classified as “likely causal” (Supporting Information) with previous reports indicating pathogenicity for late onset distal myopathy (Fiorillo et al. 2016) and the other 17 were of “uncertain significance” (Table S5). The relevance of these results will only become apparent when similar analyses are undertaken with larger samples. No individuals were identified as being homozygous for any variant.

### Identification of compound heterozygous variants in all genes

Three individuals were investigated for compound heterozygosity, because they have two rare variants in the same gene. The presence of two deleterious variants that occur one on each chromosome within a gene locus could result in complete gene dysfunction, which is known as compound heterozygosity. Alternatively, two deleterious variants may exist on the same chromosome to leave one functional allele (heterozygous mutations). For one case, the aligned sequencing reads revealed that the two variants (in *PNF1*) occurred always (73x) sequentially (i.e., on the same chromosome) and thus this individual was not compound heterozygous. The compound allele has been reported in both cases and controls, and considered to be risk variants rather than highly penetrant variants (Wu et al. 2012). One individual had two variants in the *TPP1* gene, namely a splice and a missense in exon 10. The same splice but different missense (exon 11) combination has been reported to cause spinocerebellar ataxia in a Dutch family (Sun et al. 2013). Another individual had two missense variants in the *AARS* gene occurring in the aminoacylation and tRNA recognition domain. Variants in this gene have been associated with CMT (Bansagi et al. 2015) and infantile encephalopathy (Simons et al. 2015) (Table S3).

### Causative variant and methylation signature analysis

Here, we hypothesized that carriers of pathogenic variants (i.e., *C9orf72*,* SOD1*, and *TARDBP*) may exhibit hypo‐ or hypermethylation in the same gene, relative to noncarriers. Carriers were considered outliers if they lay outside of 1.5× the interquartile range of the noncarriers. We first investigated the DNA methylation signature for nine *C9orf72* HRE‐positive ALS cases (Supporting Information). Two were identified as being methylation outliers in the two probes in the CpG island upstream of the transcriptional start site and the probe nearest to the HRE (Fig. S1). Having accurate information on the length of the expansions in individuals would allow investigation of the relation of the repeat count and to methylation outlier status.

Given that the two *SOD1* and two *TARDBP* variants identified in ALS cases were considered to have the greatest evidence for ALS causality, the methylation signature (probes closest to the TSS or the variant) was assessed in each gene. Neither gene presented compelling evidence that these rare single‐nucleotide variants were associated with differential DNA methylation in blood (Figs S1 and S2). For *SOD1*, probe cg03524836 located downstream of the variant demonstrated hypermethylation in one of two carriers, whereas for *TARDBP*, there was no evidence for altered methylation for either the closest TSS probe nor the closest variant probe in the *TARDBP* carrier.

## Discussion

**Figure 2:**
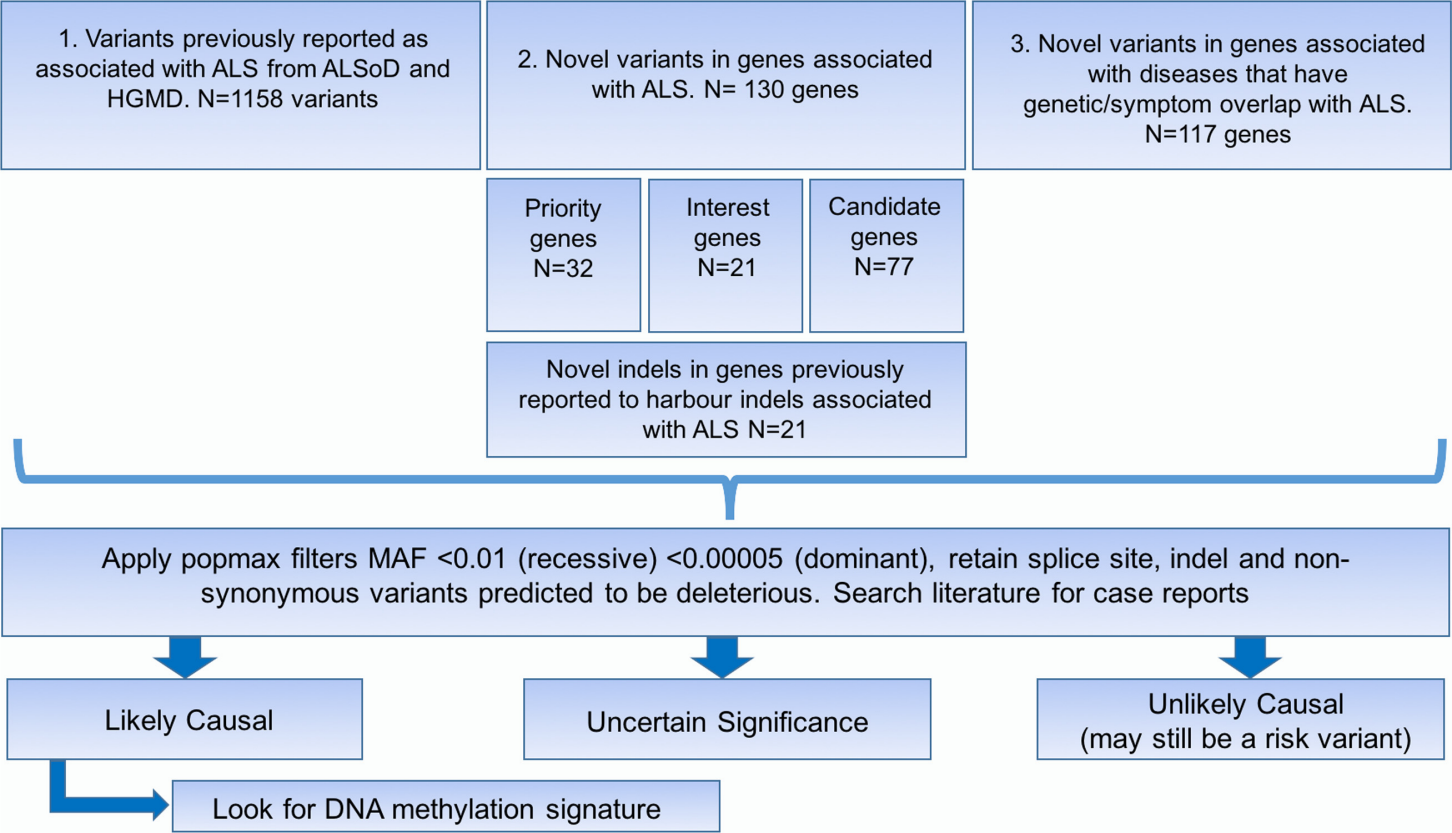
Flowchart demonstrating variant filtering methods and link with methylation data. After quality control (QC), whole exome sequences were analyzed for reported variants, novel variants in known genes, or novel variants in similar disease genes. ALS genes were divided into priority, interest, and candidate genes. This included a set of genes analyzed for the presence of insertion/deletion variants. Variants were subsequently filtered for rare, damaging variants with minor allele frequencies below the defined threshold before being prioritized based on prediction or literature. DNA methylation was collected independently and analyzed based on causal variant results. MAF, minor allele frequency.

Testing for known variants affecting ALS could assist with ALS diagnosis in a subset of those presenting to an ALS clinic. We identified four patients with previously reported *SOD1* and *TARDBP* variants, and for each the time from onset to diagnosis was 1 year. In the future, early diagnosis could have impact on clinical practice and stratification for treatment. For these individuals, variants identified have been called “likely pathogenic” using an available evidence framework (Richards et al. 2015; Li and Wang 2017). In a clinical setting, we believe reporting of such variants would be considered useful as long as presented to the patients and families with the objective interpretation through genetic counseling. Grouping of patients by pathogenic mutation could elucidate associated clinical phenotypes. To use WES as a method to screen for genetic variants in a clinic population, a critical task involves identifying relevant genes/variants and whether they are pathogenic (MacArthur et al. 2014). Here, we present a tiered strategy to identify both known variants and novel variants in ALS patients (Fig. 1) followed by an evidence‐based evaluation on whether genetic variants are likely causal or of uncertain significance (Fig. 2).

Sequencing whole exomes of our ALS case cohort has identified an additional 3% to harbor causal genetic variants – taking the overall genetic diagnosis of the cohort to 10% (accounting for the previously identified *C9orf72* HRE screening). A number of other candidate variants of uncertain significance were identified (20% ALS cases), but this list is likely to include false positives. Although there has been increasing interest in the possible role of oligogenic inheritance in ALS (i.e., more than one causative variants across more than one gene) (van Blitterswijk et al. 2012), we did not find evidence to support this in our cohort as no individual carried more than one “likely pathogenic” variant. Two individuals each carried two “uncertain significance” variants (Table S5); however, this list is likely to include false positives and thus no further conclusions can be drawn without further evidence.

**Figure 3:**
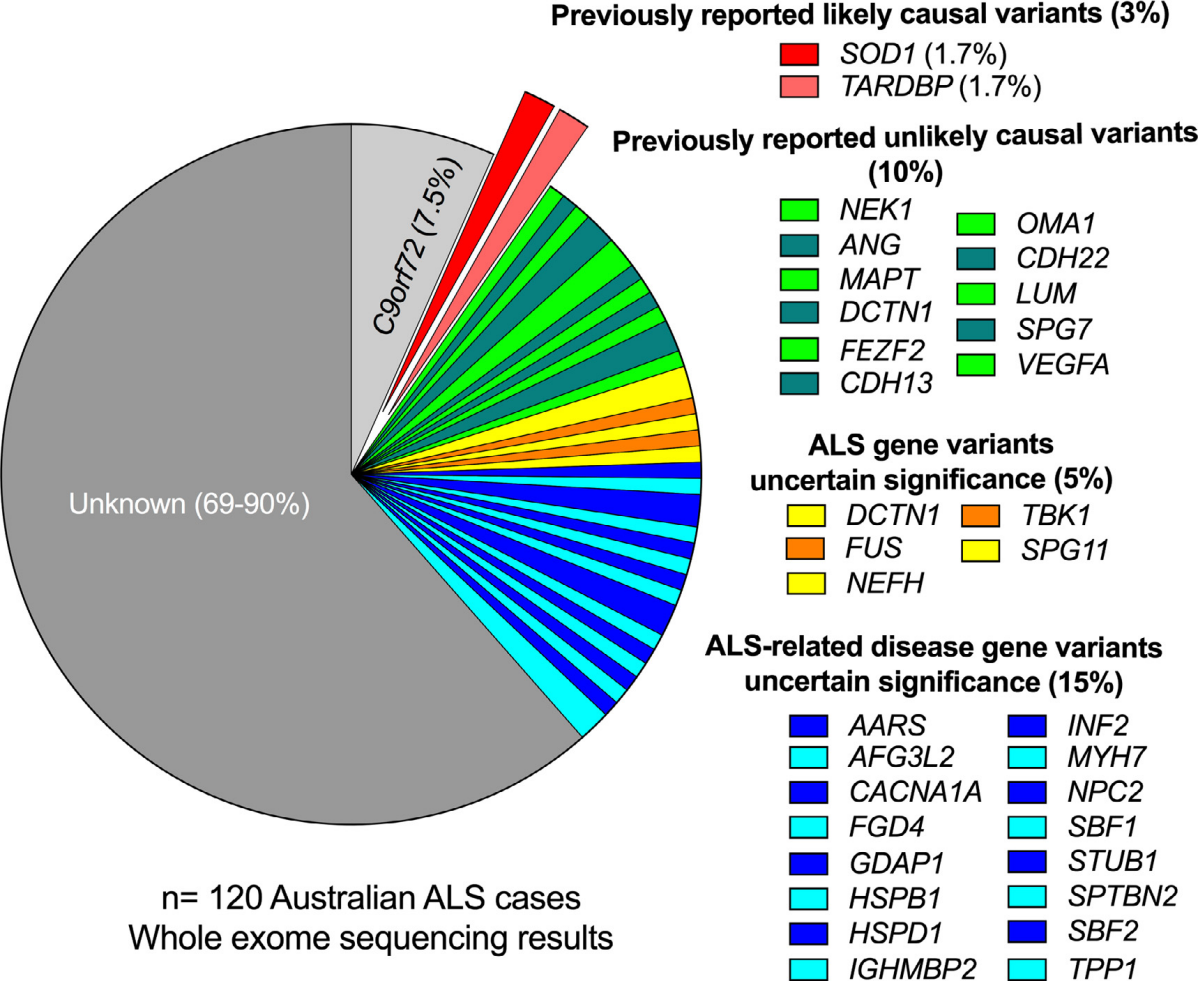
Summary of the whole exome sequencing results for Australian ALS case cohort. Variants identified were found in *n* = 1 or 2 individuals. Three percent of the cohort had a likely causal variant (exploded slices). Ten percent were positive for a previously reported ALS variant, but these were unlikely to be pathogenic because of their high population frequency. Novel variants (MAF <0.00005 dominant, <0.01 recessive), predicted to be damaging by either MetaSVM or MetaLR in ALS genes, were found in 5% of the cohort, with variants in ALS‐related disease genes being found in 15% of the cohort. Most cases did not have a known variant or novel variant in ALS genes or ALS‐related disease genes.

The WES diagnostic rate was higher when considering only fALS cases (*n* = 4/12, Supporting Information) which is consistent with other studies (McCann et al. 2017). This relatively high diagnostic rate (30%), however, does not represent a typical clinic population as familial cases consist of a small proportion presenting cases. As WES case–control sample sizes increase, better separation of true and false positives and a clearer picture of the genetic architecture of ALS (in terms of population frequencies and effect sizes of variants) will be achieved. For a late‐onset disorder such as ALS, it is relevant to note that presence of a variant in control databases does not preclude pathogenicity, however, we find, as previously reported, that many variants reported in databases as “pathogenic” occur at a population frequency too high to be consistent with the reported lifetime risk (Richards et al. 2015; Lek et al. 2016). Here, we used a population maximum frequency consistent with current guidelines (Lek et al. 2016) to classify 10% of variants as being “unlikely causal” (Fig. 3) (this does not preclude them from being risk variants).

We found variants that have been previously reported and show high evidence of causality in four patients (~3%), while we found an additional 5% and 15% of ALS patients harbor variants for which evidence is less strong (uncertain significance in ALS genes and related disease genes, respectively). In other genetic screening studies in clinic populations, variant filtering has been more lenient, typically using a MAF of 0.01 or 0.0001 (Table S6), resulting in the reporting of a greater number of identified variants, which could include a greater number of false positives. Any human population sample, whether an ALS cohort (Cirulli et al. 2015; Ozoguz et al. 2015) or other disease cohort (Lek et al. 2016), carry many rare genetic variants. If not efficiently examined, this heterogeneity can increase the difficulty and cost of genetic diagnosis, which remains a key barrier for implementation in the clinical setting (Roggenbuck et al. 2016). The use of single variant tests in clinical diagnostic laboratories is limited for ALS given the breadth of disease‐causing variants that have been identified. Sequencing a common set of well‐defined variants or genes, that is, targeted capture of the *SOD1* gene and specific exons of other genes (exon 6 of *TARDBP*, exons 14 and 15 of *FUS*), is a strategy currently being trialed (Table S6). In ALS, this portfolio of recurring mutation sites is expected to explain <5% of the cohort. With a rapidly expanding genetic profile of ALS‐associated variants (Bettencourt and Houlden 2015) the WES approach is a long‐term cost‐effective genetic testing technique and first choice of a number of studies (Kenna et al. 2013; Agarwal et al. 2016). Despite requirements for confirmation testing (in a manner that satisfies the clinic's local jurisdiction that oversee these processes), with sufficient coverage, the majority of known ALS variants can be identified using this methodology.

A notable exception to variant detection with WES is the *C9orf72* HRE, which represents a significant proportion of ALS patients in familial (38%) and sporadic cases (6%) (Majounie et al. 2012). Carriers can be identified using Southern blotting (DeJesus‐Hernandez et al. 2011b) or using a repeat‐primed PCR method (Renton et al. 2011b). More recently, a signature of extreme DNA methylation has been detected in carriers compared to controls. While the lack of sensitivity (i.e., does not identify all carriers) means that it cannot replace traditional detection methods, it does have a high specificity (i.e., if the individual has altered methylation they are likely to be a carrier) (Xi et al. 2013; He et al. 2015; Gijselinck et al. 2016). Here, we investigated if an altered methylation profile exists for other ALS variants. DNA methylation‐based approaches to disease diagnosis in ALS are yet to be fully explored or understood. Since SNPs have been associated with variation in DNA methylation levels (mQTL) (Bonder et al. 2017), we were interested to investigate whether altered methylation may also be an effect of a pathogenic protein/disease variant. The preliminary investigations reported here on *SOD1* and *TARDBP* suggest that DNA methylation signatures from blood are unlikely to help in assessing functionality or penetrance of these rare single‐nucleotide variants. Larger studies carried out in populations ideally with high frequency, likely causal variants, such as the Sardinian population for the *TARDBP* p.Ala382Thr (Orrù et al. 2012; Borghero et al. 2014), are needed to draw strong conclusions about the ability of DNA methylation outliers to help localize causal mutations and/or explain their penetrance. This includes how DNA sequence variants might alter methylation and gene expression outside the immediate location of the particular variant.

Research into the genetic basis of ALS has been rapidly advancing, identifying not only highly penetrant rare variants (our focus here, and the proportion of occurrences of ALS cases explained by such variants will become clearer as sequencing studies grow) but also an additive background of more common risk variants. Substantial variation exists in attitude and practices related to genetic testing in the clinic (Arthur et al. 2017; Vajda et al. 2017) and a consistent approach to the collection and analysis of genetic data is needed in order to build a solid framework to underpin discovery and translation to clinical practice. Understanding the variability in genetic architecture across individuals with continued resource investment (detection of both rare and common associated variants through analysis of large samples) would contribute data to the possible development of genetic score predictors to aid in early diagnosis, stratify heterogeneity in clinical trials, and help design precise treatment pathways. This should encompass both familial (10%) and sporadic cases (90%) as it is well recognized that both types of presentations have a genetic component contributing to and/or altering their ALS diagnosis and symptom presentation.

## Conclusions

We present an approach to the possible clinical use of whole exome sequencing in an Australian ALS case cohort of both familial and sporadic ALS cases. Applying this technology to a clinical cohort, we found that for 3% (four cases) it provided genetic information that could contribute to diagnosis of ALS. The *C9orf72* HRE remains the most common highly penetrant ALS‐associated variant (~7% cases in European ancestry), and while this cannot currently be identified using WES, new evidence suggests this variant can be identified by whole genome sequencing (Dolzhenko et al. 2016). The application of next‐generation sequencing to patient samples at the clinic can provide an efficient disease diagnosis for a small subset of individuals that has implications for the patient's family and in the future may guide treatment decisions tailored to the different causative variants. However, careful genetic counseling will be needed to convey the interpretation of putative pathogenic variants in those presenting without clear family history. As sample sizes for discovery of ALS‐associated loci increase, particularly with large consortium approaches such as Project MinE, we believe improved diagnostic rates and genetic risk predictors will be achieved, and a clearer picture will emerge for their overall utility in the clinical setting. Contributions of DNA methylation approaches to disease diagnosis have not been yet sufficiently investigated, but these preliminary investigations on *SOD1* and *TARDBP* did not suggest that DNA methylation signatures from blood are helpful in assessing functionality of these rare single‐nucleotide variants. Our findings present a strategic method for pathogenic variant identification, with rapid results that can contribute to further investigation on risk susceptibility and causal variants contributing to ALS.

## Conflict of Interest

None declared.

## Supporting information


**Table S1.** Gene list.
**Table S2.** Known ALS variants identified in patients that were likely causal.
**Table S3.** Previously reported ALS‐associated variants found in Brisbane ALS patients at a minor allele frequency which renders them unlikely to be causal.
**Table S4.** Novel rare damaging variants in ALS genes identified in ALS cases of unknown consequence likely comprising both true and false positives.
**Table S5.** Novel rare damaging variants in similar disease genes identified in ALS cases.
**Table S6.** Recent approaches used to genetically screen ALS patients.

**Figure S1.** Boxplots of the methylation signal in cases and controls for the *C9orf72* probes and DNA methylation levels in *C9orf72* gene region.
**Figure S2.** (A) Boxplots of the methylation signal in cases and controls for the *SOD1* probes and DNA methylation levels in the *SOD1* gene region.
**Appendix S1.** Materials, methods and results.
