# Supplementary material for: Whole exome sequencing and DNA methylation analysis in a clinical amyotrophic lateral sclerosis cohort
Source: Mol Genet Genomic Med. 2017 Jun 12;5(4):418–28. doi: 10.1002/mgg3.302 (PMC5511806; doi:10.1002/mgg3.302)
Supplement: Supplementary file 2 — Figure S1. Boxplots of the methylation signal in cases and controls for the C9orf72 probes and DNA methylation levels in C9orf72 gene region. Figure S2. (A) Boxplots of the methylation signal in cases and controls for the SOD1 probes and DNA methylation levels in the SOD1 gene region. Appendix S1. Materials, methods and results. [file MGG3-5-418-s002.docx]

**SUPPLEMENTARY MATERIAL**

**An approach to Whole Exome Sequencing and DNA methylation in a clinical Amyotrophic Lateral Sclerosis cohort**

Fleur C. Garton, Beben Benyamin, Qiongyi Zhao, Zhijun Liu, Jacob Gratten, Anjali K. Henders, Zong-Hong Zhang, Janette Edson, Sarah Furlong, Sarah Morgan, Susan Heggie, Kathryn Thorpe, Casey Pfluger, Karen A. Mather, Perminder S. Sachdev, Allan F. McRae, Matthew R. Robinson, Sonia Shah, Peter M. Visscher, Marie Mangelsdorf, Robert D. Henderson, Naomi R. Wray, Pamela A. McCombe

Corresponding authors= [naomi.wray@uq.edu.au](mailto:naomi.wray@uq.edu.au), [Pamela.McCombe@uq.edu.au](mailto:Pamela.McCombe@uq.edu.au)

Contents

Materials and methods (pages 2-5)

Results (pages 6-9)

Supplementary figures (pages 10-11)

References (pages 12-14)

MATERIALS AND METHODS

**Subjects**

In addition to patient and controls sourced from the Royal Brisbane & Women’s Hospital (RBWH) , healthy controls for the methylation capture were sourced from the Older Australian Twin Study (OATS) (N=90 twin pairs aged >60 years), (1) recruited in Brisbane Queensland Institute of Medical Research (Berghofer Medical Research Institute (QIMR)) and University of New South Wales (UNSW). Our Australian ALS case sample is ancestrally (majority) European population. The very large ExAC database (2), comprising 15 available reference populations provides the best resource to filter rare, benign variants.

**Genotyping and methylation**

*Detailed WES methods*

To prepare sequencing libraries, 500 ng of DNA (Qubit HS DNA Assay) were sheared to a mean size of 200bp using a Covaris E220. Libraries were prepared using the KAPA HYPER Prep kit (KAPA Biosystems, USA) using TruSeq illumina adapters diluted one in two. Eight indexed samples were pooled for targeted exome capture using the Nimblegen SeqCap EZ Exome v3 (64Mb) kit (Roche, USA) according to manufacturer’s instructions. Library quality was confirmed using a DNA1000 assay on the Agilent BioAnalyser, and quantitative PCR using the KAPA library quantitation kit for illumina (KAPA BioSystems, USA).

Samples were sequenced in a 126 bp paired-end mode using the Illumina HiSeq 2000 platform using v4 SBS chemistry, with each lane containing one pool (eight samples).  Image processing and sequence extraction were performed using the standard Illumina Genome Analyzer software. CASAVA (v1.8.2) was used to demultiplex the samples and generate the short reads for each individual sample in “fastq” format. The quality of raw sequencing reads was evaluated by FastQC (v0.10.1) software. In total, we generated ~741.48 Gbp of sequence data for a total number of 120 individuals that passed QC, with a mean coverage of 53.9x per individual. Sequence alignment and variants calling were performed using the BWA-Picard-GATK analysis pipeline. Paired-end reads were aligned to the human reference genome (hg19) using BWA (v0.6.2), performed the file conversion from SAM to BAM and generated the sorted and indexed BAM files using SAMtools (v0.1.17) (3), and marked duplicates using Picard software package (<http://picard.sourceforge.net>) (v1.72), and then we used GATK (v3.4-0) to perform the “Indel Realignment”, “Base Quality Score Recalibration”, “Variants Calling” (GATK HaplotypeCaller in a gVCF mode), “Joint Genotyping” and “Variant Recalibration” as instructed in GATK Best Practices (4). The analysis-ready variants from GATK analysis pipeline were annotated using the ANNOVAR software tool (version 2015 Jun17) (5). Variants were further annotated by the dbSNP database (<http://www.ncbi.nlm.nih.gov/projects/SNP/>), 1000 Genomes Project (<http://ftp.ncbi.nih.gov/>), Exome Sequencing Project (<http://evs.gs.washington.edu/EVS/)> and the Exome Aggregation Consortium (ExAC) based on minor allele frequency (MAF) polymorphisms > 1 x10^−2^ in any population (2) were removed from the analysis.

*WES Quality control*

Following sequence variant call and annotation, we performed quality control steps on individuals and variants. We used common SNPs (MAF > 1%) with high genotype call rate (> 95%) for conducting QC on individuals (N=122). Individuals were excluded from downstream analyses if i) genotyped derived sex was ambiguous or discordant from reported sex (1 individual); ii) genotyping call rate < 80% (0 individuals) iii) excessive heterozygosity rate (> 3 standard deviation from the mean of heterozygosity rate; 0 individuals); iv) genetic relationship matrix (GRM) value of > 0.1 with another individual (0 individuals). We calculated ancestry principal components (PCs) from the GRM. Individuals shown to be an ancestry outlier based on ancestry PC1 and PC2 (i.e. > 6 SD) from the mean of EUR (European) and CHB (Chinese Han from Beijing, HapMap) PCs; 7 individuals) were not excluded from the analysis but noted in files for reference. Patients were re-reviewed at time of analysis for their ALS status and removed if they presented with Frontotemporal Dementia (FTD) only (1 individual). After these QC steps, 120 cases (75 males and 45 females) remained for the WES analyses.

*WES Variant filtering and Statistical Analysis:*

Variants that were tagged as "PASS" in GATK Variant Quality Score Recalibration (VQSR) were used for downstream analysis. GATK resource bundle (v2.5) was used for VQSR, which includes known SNP sites from HapMap v3.3, the Omni2.5 array results, the phase 1 result from the 1000 Genomes Project, dbSNP v137, and the Mills and 1000G gold standard indels. The cutoffs were a tranche of 99.5% for SNPs and 99% for indels. Variants in each individual were required to have a genotype quality score (GQ) of at least 20 for further analysis.

Information from two databases were downloaded and utilised for exome filtering, the professionally curated human gene mutation database (HGMD) (trial version, accessed 3^rd^ May 2016) (6) and a curated database list of known/risk ALS variants derived from publications and direct input by researchers; the Amyotrophic Lateral Sclerosis online Database (ALSoD) (accessed 1^st^ September 2016) (7). Three reference lists were utilised 1) Known variants (ALSoD, HGMD) (n=658, n=493) 2) Known ALS genes (ALSoD, HGMD) (n=130) 3) Overlapping disease causing genes (HGMD) (fronto-temporal dementia, spinal muscular atrophy, spinal bulbar muscular atrophy, distal hereditary motor neuropathy, Charcot-Marie-Tooth disease, hereditary spastic paraplegia, hereditary ataxia, and distal myopathy (n=117) (Figure 1). To improve distinction between gene sets and variants identified in results, the genes were divided into priority, interest and candidate. The priority gene set were known ALS genes that were well recognised to contribute to ALS, the interest gene set were genes that had been reported to be associated with ALS but had not subsequently been replicated, the candidate gene set were genes that were identified in similar diseases, that had overlapping genetic or symptomatic presentations with ALS (6-8).

Results were reported in an ANNOVAR tabulated format with each search was hierarchical- i.e. variants filtered for in step 1) were ignored in step 2) and genes filtered in step 2) were ignored in step 3).

For single nucleotide variant filtering, the output from step 1) was initially filtered used reference populations from ANNOVAR to ignore common variants (MAF >0.01) and non-coding regions. We then used the literature to examine the most up to date resources available to identify rare, highly penetrant damaging variants replicated in >1 FALS or SALS case/s.

Also focusing on single nucleotide variant filtering outputs from steps 2) and 3) were filtered using a threshold of MAF in ANNOVAR reference populations (September 16^th^ 2016) (ExAC, ESP, 1000G populations) to look for autosomal dominant candidate variants (MAF <0.00005 or 0.005%) and autosomal recessive or compound heterozygous candidate variants (MAF <0.01 or 1%). To minimize false positives, we utilised a “population maximum” approach so that any variant with a minor allele frequency above the specified threshold in any reference populations would be ignored (2). Remaining variants were then filtered by removing variants in 5’UTR, 3’UTR, downstream, upstream gene regions and exonic synonymous changes. Remaining variants were non-synonymous (missense), stop-gains (nonsense) or splice sites (first and last two bases of each intron). Variants in the set of 130 ALS genes were analysed hierarchically in three stages starting with priority genes (n=32), interest genes (n=21) followed by candidate genes (n=77) (Supp. Table 1).

To identify indels, only priority genes previously published to contain indels or loss-of-function variants were considered in order to minimise the potential number of false positives (9). A list of n=21 genes (Supp. table 1) was curated from the HGMD database (6). These were prioritised if they were truncating (frame-shift), insertions and deletions.

Using available meta-analysed collapsed prediction value of MetaLR and MetaSVM (10) in ANNOVAR variants were prioritised if they were considered ‘deleterious’ by either of these metrics. MetaLR and MetaSVM are a combined evaluation of the predictive performance of eighteen current deleteriousness-scoring methods, including eleven function prediction scores (PolyPhen-2, SIFT, MutationTaster, Mutation Assessor, FATHMM, LRT, PANTHER, PhD-SNP, SNAP, SNPs&GO, and MutPred), three conservation scores (GERP++, SiPhy and PhyloP) and four ensemble scores (CADD, PON-P, KGGSeq and CONDEL). The two ensemble scores, MetaSVM and MetaLR, use Support Vector Machine (SVM) and Logistic Regression (LR) respectively, to integrate prediction scores. Rare heterozygous, homozygous and compound heterozygous variants were defined, and predicted ‘deleterious’ mutations by MetaSVM and/or MetaLR were retained. It has previously been reported that the use of consistent deleterious prediction in multiple programs can enhance pathogenicity call rates for *SOD1* mutations in ALS (11), with MetaSVM and MetaLR outperforming other programs on deleterious prediction (10).

Final variant lists were cross-checked with clinical databases (OMIM, Clinvar) and the literature for case reports to apply an available evidence framework (12, 13) to assess pathogenicity.

**DNA methylation data**

*DNA Methylation profiling*

Genomic DNA from each sample was bisulfite-converted using the EZ-96 DNA Methylation kit (Zymo Research). The placement of each DNA sample into 96 well plates was random and genome-wide methylation levels were measured using the Illumina HumanMethylation450. This methylation array contains 485,577 probes covering 99% of Reference Sequences (RefSeq) genes and 96% of CpG islands. Cases (n=120) and controls (n=111) were subject to the following stringent data quality control (QC) steps. Beta values were generated from raw intensity data after background correction and internal control normalisation using R minfi package (14) and then included filters on methylation probes and individual samples. Probes were excluded from the analyses based on the following exclusion criteria: *i*) bound to multiple locations (N: 40,650); *ii*) located at or bound to sex chromosomes (N: 12,388); *iii*) call rate < 0.95 based on a detection p-value cut off 0.01 (N=1,123). Remaining in the analysis were 432,216 clean probes. For individual quality controls, cases were removed who met the following excluding criteria: *i*) had a call rate < 95% (N: 1); *ii*) duplicate individuals based on genetic relationship calculated from 65 SNPs detected in methylation data (N: 1). Following QC, 118 cases and 111 controls were used for methylation analyses.

***C9orf72* carrier detection**

Patients diagnosed with ALS were screened for *C9orf72* HRE using a combined approach of regular PCR (PCR) and repeat-primed PCR (RP-PCR) using DNA extracted from blood (15). Samples were tested initially on PCR to amplify GC-rich fragments across the repeat. Amplified fragments electrophoresed on a 2% agarose in 1X Sodium Borate buffer identified 92 samples that did not carry an expansion (i.e. were heterozygous for allele size <~25 repeats) and 28 samples that required further testing (i.e. were homozygous for normal alleles or were carriers). RP-PCR, using a tagged primer (16) with fragments run on either southern blot gels or run on an ABI PRISM 377 DNA sequencer (Applied Biosystems, USA) to detect the presence of additional bands. This identified n=9 HRE carriers (heterozygous for >30 repeats) (7.5%) and n=19 non-carriers (homozygous for normal allele size). This primer set demonstrated good sensitivity and specificity (17). Eight *C9orf72* carriers have genome-wide SNP data (Benyamin et al., submitted) and we found 100% concordance that all *C9orf72* HRE carriers were also identified as carriers of either one (n=6, AG) or two risk alleles (n=2, AA) rs3849942 (A/G) (16).

RESULTS

## Clinical characteristics of the four ALS individuals harbouring known ALS variants that can be considered causal.

The two cases harbouring *SOD1* mutations were independently confirmed variants at a National Association of Testing Authorities (NATA) accredited laboratory. This is the clinical confirmation testing required in Australia which involves Sanger sequencing in a laboratory with specific sample handling and processing standards.

The two cases were unrelated males but both had an immediate family member that had been diagnosed with ALS. The carrier of p.Gly94Val presented with lower limb weakness aged 43 years (BMI of 36) and was diagnosed 1 year later (LMN Predominant) (MND_BIO_220). He had slow progressing ALS, was on riluzole and passed away aged 60 years, surviving 17 years with ALS. Mutations at this base encompassing all potential missense changes (i.e. glycine > alanine/cysteine/aspartic acid/arginine/valine) are suggested to impact the apex of a tight hairpin turn (joining two strands of the SOD1 monomer chain) where substitution of anything longer than the single hydrogen of glycine interferes with the correct relative positioning of the strands (18). For the p.Gly94Val variant, both a family (n=3, 46 years average age of onset, 5-7 year survival) and single patient (with a family history) have been reported (7, 19). The single patient had a younger age of onset (37 years) and presented with arm weakness and was still alive at reporting.

The second *SOD1* carrier, MND_BIO_374 harbours the variant p.Ile114Thr. He has a lower limb onset and presented aged 48 years and was diagnosed 1.5 years later. His father had ALS, he was on riluzole with BMI of 25 and is still alive (~3 years with ALS). Five patients with the same variant have been previously reported before (4 females and 1 male from Canada and the UK), three of which report a known family history (19, 20). The four females presented at age 47 (leg onset), 48, 49 and 63 (arm onset). The male presented at age 40 (leg onset). Despite both *SOD1* variants predicted to reduce protein stability but not other aspects of *SOD1* function (aggregation tendency, amyloid propensity or chaperone binding tendency) (11) both demonstrated heterogeneity (onset location and age) between affected carriers.

The two *TARDBP* carriers both had variants in the commonly reported ALS ‘hotspot’ exon 6, without a known family history. One was independently confirmed at a NATA accredited laboratory with direct sequencing. The other individual (MND_BIO_305) has not yet been recontacted for independent confirmation testing but exome sequencing shows high coverage of the region (≥50 reads per allele).

The first is female of Asian ethnicity (MND_BIO_305) carrying the p.Met337Val variant, presented with bulbar onset aged 41 years, Creatine Kinase (CK) within the normal range and a BMI of 22. She was diagnosed one year later, was not taking riluzole and is still alive today (~8 years with ALS). Twelve other patients have been recorded with the same variant in five independent studies, of both Japanese and European ancestry with an age of onset range for females (38-57 years) and males (44-57 years) each reporting a family history. The Japanese family (three cases) presented with dysarthria, bulbar onset with the European cases recorded four with lower limb onset, three had bulbar and two had cervical/spinal (21-25). This variant has been modelled both *in-vitro* and *in-vivo*, including in a transgenic mouse which demonstrates neurological defects compared to wild-type *TARDBP* (26).

The other *TARDBP* variant carrier, p.Ala382Thr (MND_BIO_375) was a European-ancestry male aged 61 years presenting with lower limb onset. He was diagnosed 3 years later with predominantly Upper Motor Neurone weakness. He had a BMI of 30, no known family history and began taking rilzuole 2 years ago, remains alive today (~5 years ALS). In the ALSoD database, 20 other patients have been reported with the same variant (five females, 15 males) with six having a family history. Patients were of French or Italian background with an age of onset for females of 44-68 years and males 32-69 years (21, 27-31). Fourteen had spinal onset, three had arm onset and two had bulbar with one unknown. ALS patients of Sardinian, Italian ancestry have a high frequency of the p.Ala382Thr variant (likely due to a founder effect) (32), with 86 out of the 366 ALS cases (23.5%) carrying the variant, compared to eight of the 700 controls (1.1% of control cohort, aged 60- 86 years) (33). The penetrance of this variant at 70 years was found to be 66.1% (95% c.i. 48.3–83.9) among women and 80.4% (95% c.i., 70.0–90.8) among men. Similar to the reports in the ALSoD database, onset location of the p.Ala382Thr presenting cases in Sardinia were predominantly spinal (80%) compared to bulbar (20%) (32).

Recent data from cell assays has demonstrated altered mitochondria function which was particularly evident in TARDBP (p.Ala382Thr) fibroblasts but not C9orf72 fibroblasts where there was an increase of mitochondrial DNA content and mass (34, 35).

For known causal variants, there remains significant heterogeneity between cases in terms of age and location of onset despite having an identical penetrant variant within the same gene. The *SOD1* had a range of onset ~25 years with weakness typically lower limb or arm based. The two *TARDBP* variants also had a large range of age of on onset (~30 years) with onset in lower limb, arm or bulbar. Thus there appears to be large variance that is not accounted for by a single penetrant variant. While functional assay work for each variant remains in preliminary stages, this type of mechanistic understanding may provide greatest promise for slowing symptoms, irrespective of when and how they present.

## WES variants identified in reference list 2: ALS genes

A number of rare, predicted damaging variants were identified in known ALS genes. Six were identified in ‘priority’ genes, three in ‘interest’ genes and seven in ‘candidate’ genes (Supp. table 4). These variants have uncertain significance and require further case reports and experimental evidence before their relationship with ALS can be defined. The variants found in ALS priority genes are briefly discussed below.

Two variants were identified in *DCTN1* (p.Asp748Glu and p.Val496Leu). Evidence for the involvement of this gene in neurodegeneration are the linkage studies for parkinsonian disorder Perry Syndrome (MIM#168605) and distal heredity motor neuropathy (DHMN) (MIM#607641). The identified variants for both conditions are located in the projecting arm of the protein, responsible for microtubule binding (CAP-glycine domain of exon 2) (36). The rare variants in this gene linked with ALS (37, 38) (MAF <0.00005) span across the protein to encompass the microtubule binding, motor binding and coil-coil domains (p.Gly59Ser, p.Gly59Arg, p.Met571Thr, p.Arg997Trp, p.Arg1101Lys). From the two patients in ALSoD (p.Arg1101Lys), they had an onset of 48-61 years, with a family history and onset in the limb or unknown. The two patients in our cohort were both European-ancestry females with bulbar onset, one aged 66 years and the other 74 years.

Variants in *FUS* have been reported in both fALS and sALS with many at the N terminus end (MIM#608030). The closest variants to the missense variant (p.Gly34Glu) identified in our patient population in exon 3 is a deletion (39) followed by a missense mutation p.Gly187Ala (40). This carrier was 79 years with bulbar onset and a family history. Our patient was a European-ancestry female, aged 72 years with bulbar onset and survived one-year post onset.

*NEFH* variants reported in ALS have been deletions (41) or insertions (42) (MIM#105400) which is in contrast to our patient identified with a missense change. The male European-ancestry patient in our cohort was diagnosed at 63 years with bulbar onset that was upper motor neurone predominant (p.Val726Leu).

In a similar manner, changes in *SPG11* are typically loss of function and/or compound heterozygous, rather than a single missense variant. The published recessive variants typically have a reported onset of 7-23 years, either bulbar or limb, which is very slowly progressing, with a mean disease duration of 34 years (MIM#602099). Mutations have been detected in Italian, Brazilian, Canadian, Japanese and Turkish families. The carrier identified in our cohort was a non- European-ancestry male with a slow progressing ALS, with an onset of ~58 years and was still alive >20 years (p.Asp1519Glu).

One patient was identified as a carrier of a *TBK1* frameshift mutation (p.Leu277fs). She was a European-ancestry diagnosed aged 72 with lower limb onset and had no family history. Five separate frameshift mutations have been reported in a European population (43) with multiple affected individuals. In the variants closest to our patient (p.T77WfsX4 and p.Y185X) the range of onset was 35-58 years with family history but without reported onset detail.

For recessive variants, one individual was homozygous for *AR* (Chr X) (MND_BIO_343). Expansions of a ‘CAG’ repeat in this gene cause spinal bulbar muscular atrophy (MIM# 313200), however other missense mutations have not previously been associated with neurodegeneration. The exon 6, p.Arg787Gln variant identified has not been reported before, and may be associated with androgen insensitivity syndrome or prostate cancer rather than ALS (44). The male patient was diagnosed at age 65 with lower limb onset and no family history. He survived 2 years 3 months following symptom onset.

## WES variants identified in reference list 3: genes associated with ‘related’ diseases

The widening clinical spectrum of genes identified in both ALS and other disorders, meant that it was appropriate to examine known genes from related diseases. Eighteen rare variants were picked up in our cohort with one (*MYH7* variant) identified in Clinvar (Supp. Table 5).

The *MYH7* variant p.Arg1434Cys identified in one case, occurs in the same exon (exon 10, p.S1435P) in which a three members of a family had late onset distal myopathy (onset age of 36yrs-70yrs) (45). The carrier in our cohort was a European-ancestry male aged 79 years who survived 6 months after onset.

For the other variants of unknown significance (n=17), eight occurred in genes that were associated with disease in a homozygous state, five were in regions of the gene that hadn’t previously been associated with disease and the remaining four were considered candidate variants given they occurred in genetic architecture previously associated with disease, in a domain or region where other causative variants in similar diseases had been reported.

No individuals were identified as being homozygous for any variant however two potential compound heterozygous individuals were identified (Supp. Table 5). One individual had a splicing variant (pathogenic for Spinocerebellar Ataxia/Ceroid lipofuscinosis when recessive) and also missense predicted damaging variant in exon10 of the *TPP1* gene. *TPP1* variants are associated with Spinocerebellar Ataxia (reduced enzyme activity) and a more severe phenotype of ceroid lipofuscinosis (loss of enzyme function). Compound heterozygous mutations have been reported which cause Spinocerebellar Ataxia (MIM#609270), including a splice-site and a missense mutation, which segregated with disease (46). Our patient had lower limb onset ALS, presented at age 69 and survived 2 years months. The *AARS* variants (p.Arg326Gln and p.Pro234Ser) were both missense with one (p.Arg326Gln) that occurred in close proximity to other missense variants that has previously been associated with CMT (p.Arg329His) (MIM#613287). The carrier in our cohort was a European-ancestry female, aged 74 years with Bulbar onset and survived two years post diagnosis.

## Familial cases and variant identification

Patients presenting with a family history (reported/suspected) have reported to have a greater diagnostic rate than sporadic patients. Here we describe whether a pathogenic variant was identified in familial patients (fALS). The patient cohort presenting to the clinic included 12 with a known/suspected family history of ALS or FTD (Table 1). Ten (8.3%) reported a first degree relative (parent/sibling) and two with a second or third degree relative (grandparent/avuncular/cousin) (1.6%) with ALS or FTD. Four of these cases were identified with a likely causal variant. This consisted of two *C9orf72* HRE cases, one *SOD1* case and *TARDBP* case, to represent a diagnostic rate of 33% (n=4/12). Considering variants identified from exome sequencing alone, the diagnostic rate was 16.7% (n=2/12). Another five reported a family history of dementia, multiple sclerosis or parkinsonism. None of these patients were identified with known causal variants.

Supplementary figure 1.


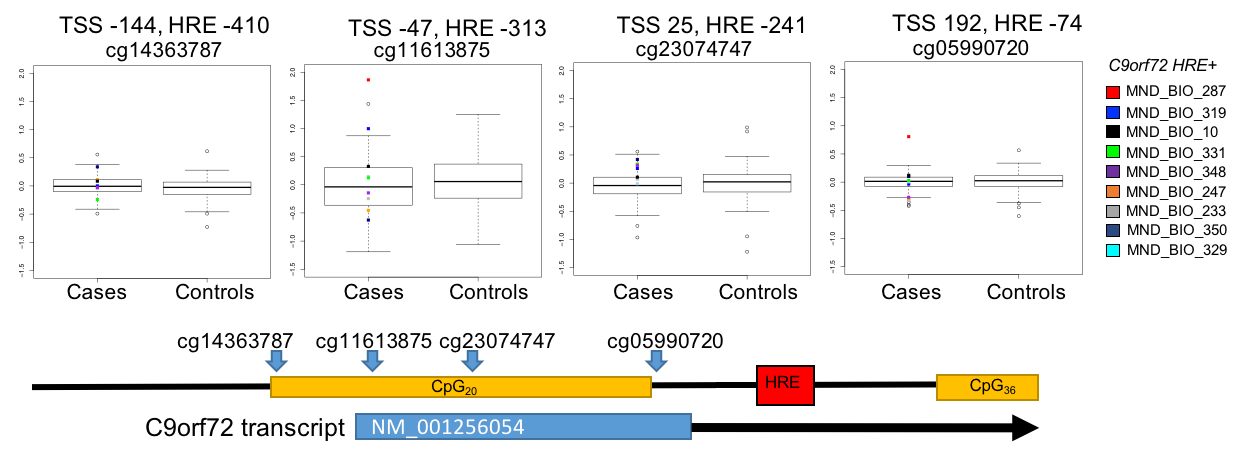


Supplementary Figure 1. Boxplots of the methylation signal in cases and controls for the *C9orf72* probes and DNA methylation levels in *C9orf72* gene region. The boxes show individuals with beta values between the first and third quartiles, the black horizontal line shows the median methylation value in cases and controls, and outliers (methylation values >1·5 times interquartile range) are shown as black circles. The methylation level in the 9 individuals with repeat expansion is indicated in the legend. The distance of the CpG probe to the transcription start site (TSS) and the hexanucleotide expansion repeat (HRE) is given above each figure. A map demonstrates the interval between the HRE, CpG islands and the methylation probes (adapted from Xi, Zinman, Moreno, Schymick, Liang, Sato, Zheng, Ghani, Dib, Keith, Robertson and Rogaeva (47) and He, et al. (48)). From previously identified probes, two probes demonstrated hypermethylation outliers in 2/9 carriers (cg05990720 and cg11613875).

Supplementary figure 2.


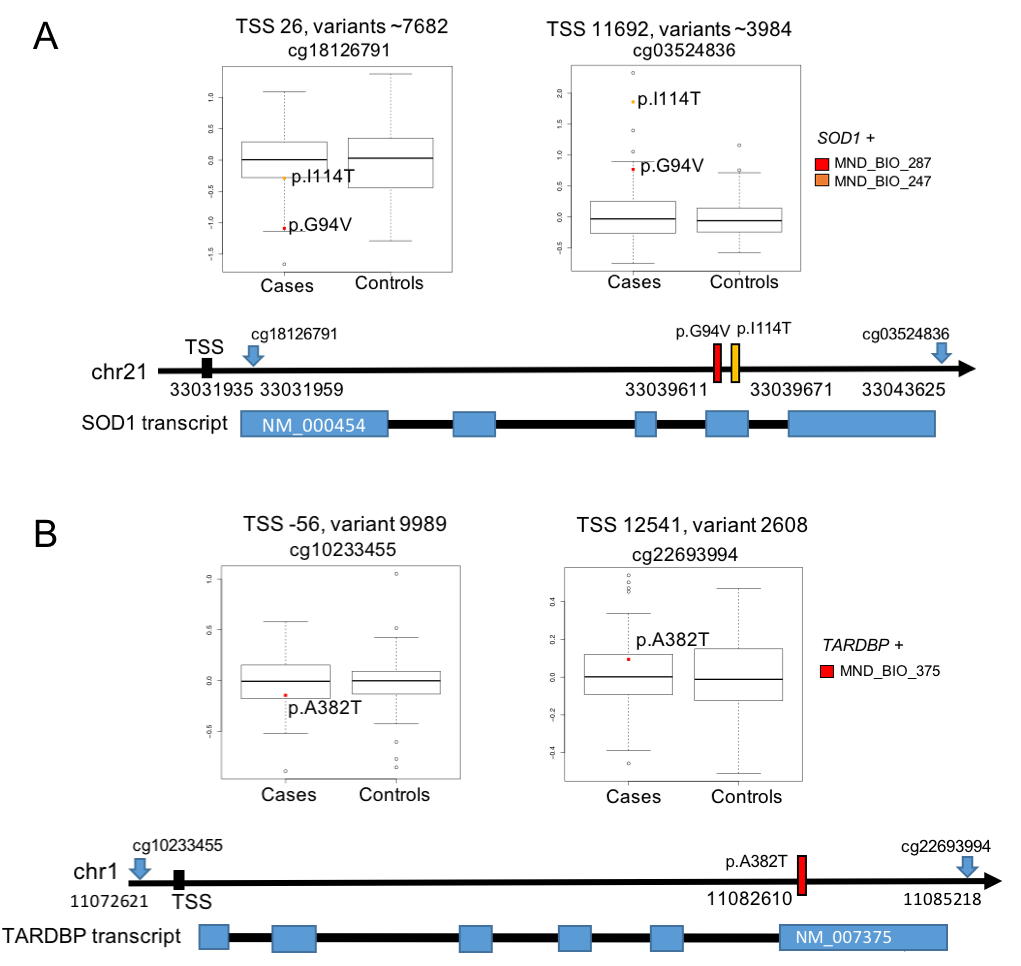


Supplementary Figure 2. **A.** Boxplots of the methylation signal in cases and controls for the *SOD1* probes and DNA methylation levels in the *SOD1* gene region. The boxes show individuals with beta values between the first and third quartiles, the black horizontal line shows the median methylation value in cases and controls, and outliers (methylation values >1·5 times interquartile range) are shown as black circles. The methylation level for the 2 SOD1 positive individuals are highlighted in red and orange as indicated in the legend. The base pair distance of the probe to the transcription start site (TSS) and the variant is given above each figure. A map (not to scale) demonstrates the interval between the TSS, variants and the methylation probes (GRCh37/hg19). In this study, only 1 probe demonstrated hypermethylation outliers in 1/2 carriers (cg03524836). **B.** Box plots for the TARDBP methylation signal in cases and controls for the *TARDBP* probes and DNA methylation levels in the *TARDBP* gene region. The methylation level in 1 individual with a TARDBP variant is highlighted in red. The base pair distance of the probe to the transcription start site (TSS) and the variant is given above each figure. A map (not to scale) demonstrates the intervals between the TSS, variants and the methylation probes (GRCh37/hg19). No probe had altered methylation in 1 carrier examined.

Supplementary References

1. Sachdev PS, Lammel A, Trollor JN et al. A comprehensive neuropsychiatric study of elderly twins: the Older Australian Twins Study. Twin research and human genetics : the official journal of the International Society for Twin Studies 2009: 12: 573-582.

2. Lek M, Karczewski KJ, Minikel EV et al. Analysis of protein-coding genetic variation in 60,706 humans. Nature 2016: 536: 285-291.

3. Li H, Handsaker B, Wysoker A et al. The Sequence Alignment/Map format and SAMtools. Bioinformatics (Oxford, England) 2009: 25: 2078-2079.

4. Van der Auwera GA, Carneiro MO, Hartl C et al. From FastQ data to high confidence variant calls: the Genome Analysis Toolkit best practices pipeline. Current protocols in bioinformatics / editoral board, Andreas D Baxevanis [et al] 2013: 43: 11.10.11-33.

5. Wang K, Li M, Hakonarson H. ANNOVAR: functional annotation of genetic variants from high-throughput sequencing data. Nucleic acids research 2010: 38: e164.

6. Stenson PD, Mort M, Ball EV et al. The Human Gene Mutation Database: building a comprehensive mutation repository for clinical and molecular genetics, diagnostic testing and personalized genomic medicine. Human genetics 2014: 133: 1-9.

7. Abel O, Powell JF, Andersen PM et al. ALSoD: A user-friendly online bioinformatics tool for amyotrophic lateral sclerosis genetics. Human mutation 2012: 33: 1345-1351.

8. Abel O, Powell JF, Andersen PM et al. Credibility Analysis of Putative Disease-Causing Genes Using Bioinformatics. PloS one 2013: 8: e64899.

9. Fang H, Wu Y, Narzisi G et al. Reducing INDEL calling errors in whole genome and exome sequencing data. Genome medicine 2014: 6: 89.

10. Dong C, Wei P, Jian X et al. Comparison and integration of deleteriousness prediction methods for nonsynonymous SNVs in whole exome sequencing studies. Hum Mol Genet 2015: 24: 2125-2137.

11. Moreira LGA, Pereira LC, Drummond PR et al. Structural and Functional Analysis of Human SOD1 in Amyotrophic Lateral Sclerosis. PloS one 2013: 8: e81979.

12. Li Q, Wang K. InterVar: Clinical Interpretation of Genetic Variants by the 2015 ACMG-AMP Guidelines. The American Journal of Human Genetics 2017: 100: 267-280.

13. Richards S, Aziz N, Bale S et al. Standards and guidelines for the interpretation of sequence variants: a joint consensus recommendation of the American College of Medical Genetics and Genomics and the Association for Molecular Pathology. Genetics in medicine : official journal of the American College of Medical Genetics 2015: 17: 405-424.

14. Aryee MJ, Jaffe AE, Corrada-Bravo H et al. Minfi: a flexible and comprehensive Bioconductor package for the analysis of Infinium DNA methylation microarrays. Bioinformatics (Oxford, England) 2014: 30: 1363-1369.

15. Renton AE, Majounie E, Waite A et al. A hexanucleotide repeat expansion in C9ORF72 is the cause of chromosome 9p21-linked ALS-FTD. Neuron 2011: 72.

16. DeJesus-Hernandez M, Mackenzie IR, Boeve BF et al. Expanded GGGGCC hexanucleotide repeat in non-coding region of C9ORF72 causes chromosome 9p-linked frontotemporal dementia and amyotrophic lateral sclerosis. Neuron 2011: 72: 245-256.

17. Akimoto C, Volk AE, Blitterswijk M et al. A blinded international study on the reliability of genetic testing for GGGGCC-repeat expansions in C9orf72 reveals marked differences in results among 14 laboratories. Journal of medical genetics 2014: 51.

18. Hosler BA, Nicholson GA, Sapp PC et al. Three novel mutations and two variants in the gene for Cu/Zn superoxide dismutase in familial amyotrophic lateral sclerosis. Neuromuscul Dis 1996: 6: 361-366.

19. Brown JA, Min J, Staropoli JF et al. SOD1, ANG, TARDBP and FUS mutations in amyotrophic lateral sclerosis: a United States clinical testing lab experience. Amyotrophic lateral sclerosis : official publication of the World Federation of Neurology Research Group on Motor Neuron Diseases 2012: 13: 217-222.

20. Rosen DR, Siddique T, Patterson D et al. Mutations in Cu/Zn superoxide dismutase gene are associated with familial amyotrophic lateral sclerosis. Nature 1993: 362: 59-62.

21. Corrado L, Ratti A, Gellera C et al. High frequency of TARDBP gene mutations in Italian patients with amyotrophic lateral sclerosis. Human mutation 2009: 30: 688-694.

22. Rutherford NJ, Zhang YJ, Baker M et al. Novel mutations in TARDBP (TDP-43) in patients with familial amyotrophic lateral sclerosis. PLoS genetics 2008: 4: e1000193.

23. Sreedharan J, Blair IP, Tripathi VB et al. TDP-43 mutations in familial and sporadic amyotrophic lateral sclerosis. Science (New York, NY) 2008: 319: 1668-1672.

24. Kirby J, Goodall EF, Smith W et al. Broad clinical phenotypes associated with TAR-DNA binding protein (TARDBP) mutations in amyotrophic lateral sclerosis. Neurogenetics 2010: 11: 217-225.

25. Tamaoka A, Arai M, Itokawa M et al. TDP-43 M337V mutation in familial amyotrophic lateral sclerosis in Japan. Internal medicine (Tokyo, Japan) 2010: 49: 331-334.

26. Janssens J, Wils H, Kleinberger G et al. Overexpression of ALS-associated p.M337V human TDP-43 in mice worsens disease features compared to wild-type human TDP-43 mice. Molecular neurobiology 2013: 48: 22-35.

27. Daoud H, Valdmanis PN, Kabashi E et al. Contribution of TARDBP mutations to sporadic amyotrophic lateral sclerosis. Journal of medical genetics 2009: 46: 112-114.

28. Kabashi E, Valdmanis PN, Dion P et al. TARDBP mutations in individuals with sporadic and familial amyotrophic lateral sclerosis. Nature genetics 2008: 40: 572-574.

29. Del Bo R, Ghezzi S, Corti S et al. TARDBP (TDP-43) sequence analysis in patients with familial and sporadic ALS: identification of two novel mutations. European journal of neurology 2009: 16: 727-732.

30. Conforti FL, Sproviero W, Simone IL et al. TARDBP gene mutations in south Italian patients with amyotrophic lateral sclerosis. Journal of neurology, neurosurgery, and psychiatry 2011: 82: 587-588.

31. Bertolin C, D'Ascenzo C, Querin G et al. Improving the knowledge of amyotrophic lateral sclerosis genetics: novel SOD1 and FUS variants. Neurobiology of aging 2014: 35: 1212.e1217-1212.e1210.

32. Orrù S, Manolakos E, Orrù N et al. High frequency of the TARDBP p.Ala382Thr mutation in Sardinian patients with amyotrophic lateral sclerosis. Clinical genetics 2012: 81: 172-178.

33. Borghero G, Pugliatti M, Marrosu F et al. Genetic architecture of ALS in Sardinia. Neurobiology of aging 2014: 35: 2882.e2887-2882.e2812.

34. Raman R, Allen SP, Goodall EF, Kramer S, Ponger LL, Heath PR, Milo M, Hollinger HC, Walsh T, Highley JR et al. Gene expression signatures in motor neurone disease fibroblasts reveal dysregulation of metabolism, hypoxia-response and RNA processing functions. Neuropathol Appl Neurobiol. 2015;41:201–26. doi:10.1111/nan.12147.

35. Onesto E, Colombrita C, Gumina V et al. Gene-specific mitochondria dysfunctions in human TARDBP and C9ORF72 fibroblasts. Acta Neuropathol Commun 2016: 4: 47.

36. Farrer MJ, Hulihan MM, Kachergus JM et al. DCTN1 mutations in Perry syndrome. Nature Genet 2009: 41: 163-165.

37. Munch C, Sedlmeier R, Meyer T et al. Point mutations of the p150 subunit of dynactin (DCTN1) gene in ALS. Neurology 2004: 63: 724-726.

38. Daoud H, Valdmanis PN, Gros-Louis F et al. Resequencing of 29 candidate genes in patients with familial and sporadic amyotrophic lateral sclerosis. Arch Neurol 2011: 68: 587-593.

39. Belzil VV, Valdmanis PN, Dion PA et al. Mutations in FUS cause FALS and SALS in French and French Canadian populations. Neurology 2009: 73: 1176-1179.

40. Rademakers R, Stewart H, Dejesus-Hernandez M et al. Fus gene mutations in familial and sporadic amyotrophic lateral sclerosis. Muscle & nerve 2010: 42: 170-176.

41. Al-Chalabi A, Andersen PM, Nilsson P et al. Deletions of the heavy neurofilament subunit tail in amyotrophic lateral sclerosis. Hum Mol Genet 1999: 8: 157-164.

42. Tomkins J, Usher P, Slade JY et al. Novel insertion in the KSP region of the neurofilament heavy gene in amyotrophic lateral sclerosis (ALS). Neuroreport 1998: 9: 3967-3970.

43. Freischmidt A, Wieland T, Richter B et al. Haploinsufficiency of TBK1 causes familial ALS and fronto-temporal dementia. Nature neuroscience 2015: 18: 631-636.

44. Gottlieb B, Beitel LK, Nadarajah A et al. The androgen receptor gene mutations database: 2012 update. Human mutation 2012: 33: 887-894.

45. Fiorillo C, Astrea G, Savarese M et al. MYH7-related myopathies: clinical, histopathological and imaging findings in a cohort of Italian patients. Orphanet J Rare Dis 2016: 11: 91.

46. Sun Y, Almomani R, Breedveld GJ et al. Autosomal recessive spinocerebellar ataxia 7 (SCAR7) is caused by variants in TPP1, the gene involved in classic late-infantile neuronal ceroid lipofuscinosis 2 disease (CLN2 disease). Human mutation 2013: 34: 706-713.

47. Xi Z, Zinman L, Moreno D et al. Hypermethylation of the CpG Island Near the G(4)C(2) Repeat in ALS with a C9orf72 Expansion. Am J Hum Genet 2013: 92: 981-989.

48. He J, Tang L, Benyamin B et al. C9orf72 hexanucleotide repeat expansions in Chinese sporadic amyotrophic lateral sclerosis. Neurobiology of aging 2015: 36: 2660.e2661-2660.e2668.
